# Un caso particular de un schwannoma en el pie

**DOI:** 10.1016/j.aprim.2025.103351

**Published:** 2025-08-08

**Authors:** Carlos Jiménez Martínez

**Affiliations:** a Servicio de Medicina Familiar y Comunitaria, Centro de salud Montesa, SERMAS, Madrid, España; b Facultad de Medicina, Universidad Francisco de Vitoria, Madrid, España

Se describe el caso de una mujer de 80 años sin antecedentes de interés que acude a la consulta por presentar un nódulo en el dorso del pie izquierdo desde hace unos 2 años. Refiere que en los últimos meses tiene dolor y parestesias en zonas distales a la tumoración.

A la exploración física presenta un nódulo de localización anterior a la articulación tibioperoneoastragalina, no doloroso, no adherido a planos profundos, de aproximadamente un centímetro de diámetro. No se objetivan otros hallazgos relevantes.

Se solicita ecografía de partes blandas en la que describen un nódulo hipoecogénico con fina cápsula y escasa vascularización distal, compatible con un angioleiomioma. No es posible descartar otras opciones (fig. 1 y fig. 2).

**Figura 1:**
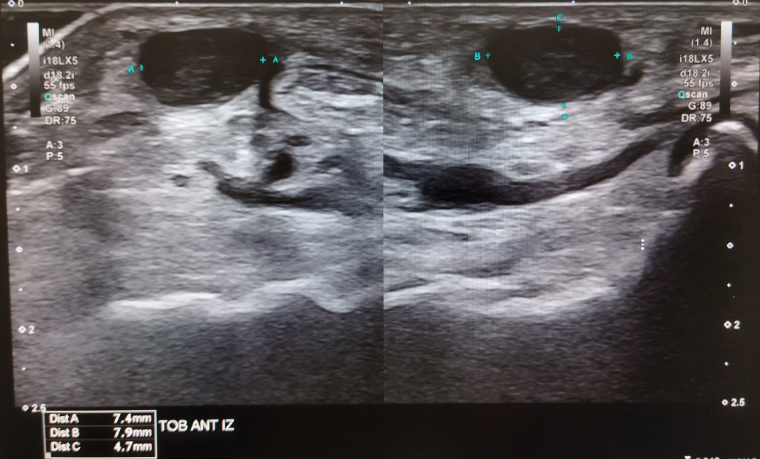
Corte axial y longitudinal ecográfico del dorso del pie derecho en modo B.

**Figura 2:**
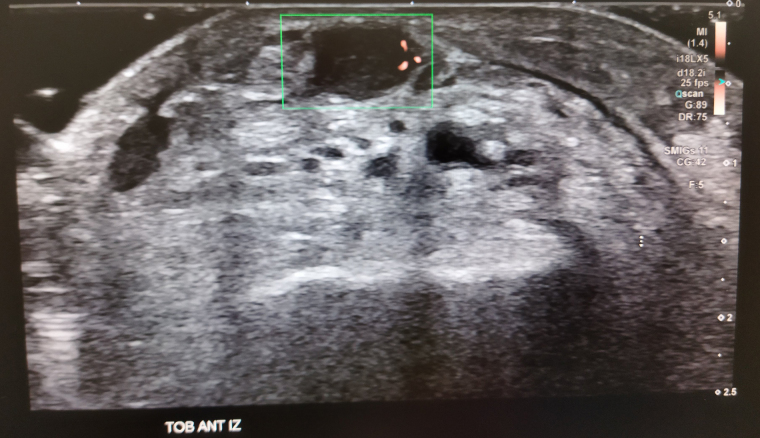
Corte axial ecográfico del dorso del pie derecho en modo Doppler color.

Se realiza interconsulta a traumatología. Tras extirpar la lesión, obtienen un fragmento de superficie lisa y brillante de 0,9 × 0,6 × 0,6 mm de consistencia elástica rodeado por una cápsula. El diagnóstico anatomopatológico fue de un schwannoma.

Un schwannoma es un tumor benigno del nervio periférico constituido por células de Schwann que suele desarrollarse en el tejido subcutáneo de la cabeza, cuello y extremidades. Presenta la misma incidencia en ambos sexos y resulta más habitual entre la cuarta y la sexta décadas de la vida^1^.

La particularidad de este caso es que la aparición de un schwannoma en el pie es muy infrecuente: tan solo entre el 4 y el 10% de los tumores tienen esta localización^2^.

Lo usual es que sea una tumoración de crecimiento lento. La sintomatología depende de las estructuras que comprime, por lo que la mayor parte son asintomáticos durante años^3^.

El diagnóstico de aproximación se tiende a realizar mediante ecografía, en la que, característicamente, se aprecia una lesión sólida redondeada, bien delimitada e hipoecoica. Si bien suele ser más útil una resonancia magnética, en la que, además, se puede estudiar su relación con los nervios y tejidos adyacentes^4^.

El tratamiento definitivo es la extirpación quirúrgica. La recurrencia es muy rara (menos del 1% de los casos)^2^.

## Financiación

Esta publicación ha sido financiada a través de la Fundación para la Investigación e Innovación Biomédica de Atención Primaria (FIIBAP).

## Conflicto de intereses

El autor declara no tener ningún conflicto de intereses.
